# A Prediction Modeling Based on the Hospital for Special Surgery (HSS) Knee Score for Poor Postoperative Functional Prognosis of Elderly Patients with Patellar Fractures

**DOI:** 10.1155/2021/6620504

**Published:** 2021-12-06

**Authors:** Chenting Ying, Chenyang Guo, Zhenlin Wang, Yiming Chen, Jiahui Sun, Xin Qi, Yisheng Chen, Jie Tao

**Affiliations:** ^1^Department of Orthopedics, Shanghai General Hospital, Shanghai Jiao Tong University School of Medicine, Shanghai, China; ^2^Department of Neurosurgery, Shanghai General Hospital, Shanghai Jiao Tong University School of Medicine, Shanghai, China; ^3^Department of Sports Medicine, Huashan Hospital, Fudan University, Shanghai, China

## Abstract

**Background:**

The main aim of this study was to develop a nomogram prediction model for poor functional prognosis after patellar fracture surgery in the elderly based on the hospital for special surgery (HSS) knee score.

**Methods:**

A retrospective analysis of 168 elderly patients with patellar fractures was performed to collect demographic data, knee imaging, and functional prognosis preoperatively and during the 6-month postoperative follow-up period. Good functional prognosis of knee joint was defined as the percentage of HSS knee scores on the injured side relative to the uninjured side ≥ 80% at six-month postoperative review. Multifactorial linear regression analysis and logistic regression analysis were then used to identify risk factors of functional prognosis and develop the nomogram prediction model. Furthermore, the validity and accuracy of the prediction model were evaluated using C-index, area under the curve (AUC), and decision curve analyses.

**Results:**

The final screening from the 12 potential risk factors yielded three high-risk factors which were included in the nomogram prediction model: advanced age (OR 0.28 (95% CI 0.11-0.67), *P* = 0.005), sarcopenia (OR 0.11 (95% CI 0.05-0.26), *P* < 0.001), and low albumin level (OR 1.14 (95% CI 1.02-1.29), *P* = 0.025). The model had a good predictive ability with an AUC of 0.857 (95% CI (0.783-0.929)) for the training group and a C-index of 0.836 for the overall sample. In addition, the decision analysis curve indicated that the model had good clinical applicability.

**Conclusion:**

Our predictive model is effective in predicting the risk of poor functional prognosis after patellar fracture surgery in the elderly by assessing high-risk factors such as advanced age, sarcopenia, and serum albumin levels. This prediction model can help clinicians to make individualized risk prediction, early identification of patients at high risk for poor functional outcome, and appropriate interventions.

## 1. Introduction

The patella, an integral part of the human knee extension device, plays an important biomechanical function in knee joint movement. Previous studies have reported that patellar fracture is a common fracture of the lower extremities which occurs mostly in the aging population, with an increasing incidence resulting from low energy trauma such as falls [1, 2]. The vast majority of patellar fractures require surgical intervention [3], and the postoperative functional recovery varies from patient to patient. Therefore, identifying the factors that influence functional prognosis can help clinicians to improve patient recovery.

Sarcopenia is a progressive and generalized skeletal muscle disorder involving the accelerated loss of muscle mass and function [4–6]. Previous studies have demonstrated an association between sarcopenia and adverse health outcomes such as falls, long-term care placement, poorer quality of life, and mortality [7–9]. In addition, epidemiological studies conducted in Asian countries using the AWGS 2014 criteria have reported that the prevalence of sarcopenia ranged from 5.5% to 25.7% [10–12]. Generally, patients admitted in hospitals and in care homes have a higher prevalence of sarcopenia when compared with people in the community. Currently, there is neglect and inadequate treatment of sarcopenia despite it being an important public health problem. In addition, the clinical significance of sarcopenia on functional prognosis after orthopedic surgery has not been elucidated.

The functional prognosis after patellar fracture surgery directly affects the patients' quality of life. Therefore, development of accurate predictive models and early individualized interventions may be effective in improving patients' functional outcomes. Previous studies have reported that the Hospital for Special Surgery knee score (HSS) is an effective and reliable instrument for evaluating the functional prognosis of patella fractures [13–15]. The main objective of this study was to develop a predictive model of poor functional prognosis after patellar fracture in the elderly based on HSS scores, with the overarching goal of exploring the risk factors associated with functional prognosis.

## 2. Materials and Methods

### 2.1. Setting and Sample

**Figure 1:**
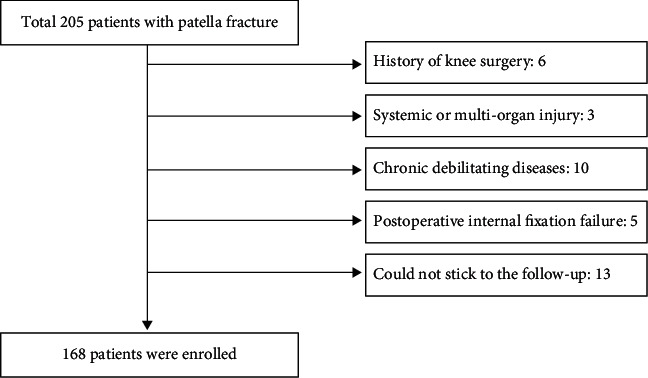
Flow chart for screening patients.

This retrospective study was approved by the Ethics Committee of Shanghai General Hospital affiliated to Shanghai Jiao Tong University (approval No. 2020KY220). In addition, the study met the stipulations of the Helsinki declaration. We have referred to this document and calculated the sample size required for this study [16]. We screened patellar fracture patients over 60 years old who were treated using Kirschner wire tension band fixation at our institution from November 2018 to May 2020. All the patients were residents of China, and the treatment procedure was performed by one orthopedic trauma surgeon with more than fifteen years of experience. Kirschner's wire and tension band were provided by Trauson (China) and Zimmer (USA), respectively. The operative criteria was as follows: patellar fractures displaced more than 2 mm, uneven joint surface over 2 mm, and fractures with extensor support band tear [17]. Patients were excluded if they had previous knee movement dysfunction, history of knee surgery, associated systemic or multiorgan injury, cognitive impairment, chronic debilitating diseases (neoplasms, renal insufficiency, and Parkinson's disease), postoperative internal fixation failure and infection, or follow-up of less than six months. Signed informed consent was obtained from the participating patients prior to starting the study. Ultimately, 168 patients, 63 males and 105 females, who met the inclusion criteria were included in this study; 37 patients were excluded according to the exclusion criteria (Figure 1). The fracture types were then classified using AO classification system according to the preoperative X-ray and three-dimensional computed tomography (3D CT) of the knee joint. The laboratory blood test results taken in this study were the patients' preoperative fasting venous blood samples. In addition, we obtained the basic clinical characteristics of the patients such as sex and age before the surgery.

### 2.2. Assessment of Sarcopenia

The definition of sarcopenia in this study was based on the consensus of the Asian Working Group for Sarcopenia (AWGS) [18, 19]. The patients were classified as having sarcopenia if they had low muscle strength and low muscle mass. The body weight adjusted cross-sectional area of midthigh muscle (tmCSA/BW cm^2^/kg) measured by CT (Siemens, Germany) was used as an indicator of low muscle mass (males ≤ 1.58 cm^2^/kg and females ≤ 1.25 cm^2^/kg) [18, 20]. The middle of the thigh is defined as the midpoint of the line between the midpoint of femoral intertrochanteric crest and the midpoint of intercondylar line of the femur. On the other hand, the handgrip strength is used as an indicator of low muscle strength (males < 26 kg and females < 18 kg) [19]. In this study, the grip strength was measured in the dominant hand using a Jamar ergometer (Asimow Engineering, USA) with the elbow flexed at 90 degrees and the forearm in a neutral position. The average of three trials was recorded in kilograms, with a 5-minute interval between trials. All patients completed the sarcopenia assessment before operation.

### 2.3. Functional Prognosis Measure

The Hospital for Special Surgery (HSS) knee score and the range of motion of knee joint were used to evaluate knee function [21], where the assessments were performed preoperatively, three months postoperatively, and six months postoperatively. The range of motion of knee joint was evaluated using a standard goniometer, and the final value was reported as a percentage of the injured side relative to the uninjured side. We hypothesized that the functional status of the knee on the injured side would be similar to that on the uninjured side due to the inability of accurately measuring the functional status of the knee prior to the fracture on the injured side. This was then verified using a clinical interview, and the functional status of the patient was measured as a percentage of the HSS knee scores on the injured side relative to the uninjured side. The first assessment was completed by two independent orthopedic surgeons within 24 hours of admission, and the average of the two measurements was used for the final analysis. At three and six months after the operation, the patient returned to the hospital for knee function and radiographic evaluation. Two orthopedic surgeons, who did not know the patient's clinical information, assessed the patient's knee mobility and completed the patient's HSS knee scores during this period. The average of their measurements was then used for the final analysis. The percentage of HSS knee scores on the injured side relative to the uninjured side at 6-month postoperative review ≥ 80% was used as an indicator of good functional prognosis [13].

### 2.4. Radiological Assessment

At six months postoperatively, radiological assessment was performed by two independent observers, who did not know the patient's clinical information. Insall-Salvati index [22], Blackburne-Peel index [23], congruence angle [24], and lateral patellofemoral angles [25] were measured using lateral and Laurin knee radiograph. The measurement was repeated three times, and the average of the measurements was used for the final analysis.

### 2.5. Statistical Analysis

The Kolmogorov-Smirnov test was used to determine the normality of variable distributions. Continuous data that followed a normal distribution were expressed as mean and standard deviation, while continuous data that did not follow a normal distribution were expressed as median (25% percentile–75% percentile). The independent samples *t*-test and Mann–Whitney test were used to test normally distributed continuous and nonnormally distributed continuous variables, respectively. In addition, the chi-square test was used to test the differences in categorical variables. All the statistical tests in this study were performed using Windows SPSS (Release 22.0; SPSS. Chicago, IL, USA). The categorical variables were dummy coded with the subgroup using the largest sample size as the reference group. Linear regression analysis and forward stepwise variable selection were used to initially determine the relationship between potential predictors and the dependent variables (HSS knee scores at 6 months postoperatively) [26] and performed residual analysis to determine model validity. *P* < 0.05 was considered to be statistically significant.

Principal component analysis (PCA) was used to check the distribution of the dataset. Furthermore, a multivariate logistic regression model was used to verify the potential influences identified by the multifactorial linear regression model and then used to develop a nomogram predictive model for predicting the risk of poor functional outcomes in elderly patients with patellar fractures after surgery. A calibration curve was used to assess the accuracy of nomogram [27]. We then measured the C-index and plotted the area under curve (AUC) in order to further quantify the recognition performance of the nomogram [28]. R language (R software version 3.5.3) was used to perform further iterations (10,000 repeated samples) on the nomogram to more accurately calculate the C-index [29]. Finally, decision curve analysis was used to assess the clinical utility of the nomogram [30].

## 3. Results

### 3.1. Basic Characteristics of the Data

We collected the complete data from 168 elderly patients with patella fractures (63 males and 103 females). The males had an average age of 65.75 ± 6.42 years, while the females had an average age of 66.62 ± 6.79 years. Patients were categorized into good functional prognosis and bad functional prognosis groups based on the definition of good functional prognosis in the method. The characteristics of all the patients are shown in Table 1. At 6 months postoperatively, 122 of 168 patients (72.6%) had a good functional prognosis, while the remaining 46 patients (27.4%) had a poor functional prognosis. The obtained results indicated that there were significant differences in age, cardiac disease, sarcopenia, BMI, serum albumin, and hemoglobin between the two groups (*P* < 0.05). The group with poor functional prognosis was older and had higher rates of cardiac disease and sarcopenia. In addition, they had a lower BMI, serum albumin, and hemoglobin levels.

### 3.2. Screening of Predictors

**Figure 2:**
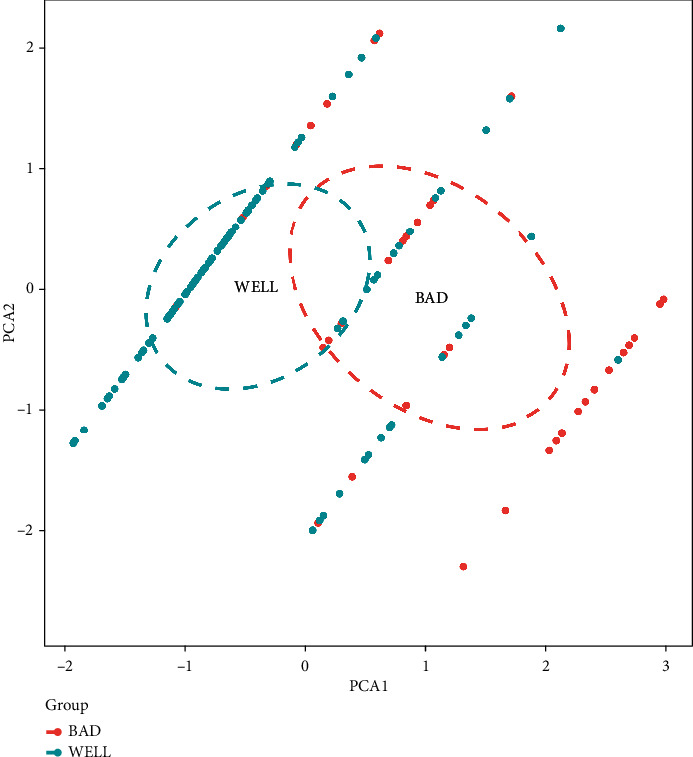
Results of principal component analysis. Blue dots represent good prognosis samples, while red dots represent poor prognosis samples.

**Figure 3:**
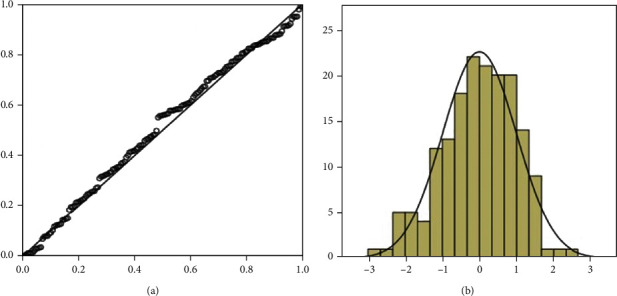
(a) P-P chart of regression-standardized residuals. (b) Histogram of residual analysis.

The obtained radiographic results during postoperative follow-up revealed that there was no significant difference between the good functional prognosis group and the poor functional prognosis group (Table 2). Moreover, the two groups did not differ statistically in the recovery of knee range of motion, but the HSS scores of the two groups were significantly different. The HSS scores of the good functional prognosis group were significantly higher than those of the poor functional prognosis group at three months and six months postoperatively (both *P* < 0.001, Table 3). The HSS score at six months postoperatively was then used as the dependent variable where single-factor, multifactor linear regression analysis, and forward stepwise variable selection were performed to initially determine the relationship between the potential risk factors and the dependent variable. Three risk factors were selected from fifteen possible influences: advanced age (Beta = −0.48, *P* < 0.001, Partial *R*^2^ = 0.405), sarcopenia (Beta = −0.42, *P* < 0.001, Partial *R*^2^ = 0.173), and hypoalbuminemia (Beta = 0.14, *P* = 0.007, Partial *R*^2^ = 0.016); the results indicated that the relative magnitude of the total regression contribution of these three factors was 59.4% (Table 4). PCA results showed that there was no multicollinearity in the data distribution; P-P chart of regression-standardized residuals and histogram of residual analysis showed that the data distribution was linear and normal distribution, respectively (Figures 2 and 3). The variance inflation factor of predictors was less than 10, and the tolerance was greater than 0.1 showed that data distribution had independence and consistency of variance (Table 4). In summary, the model had good linear trend, independence, normality, and consistency of variance.

### 3.3. Establishment and Test of the Nomogram Prediction Model

**Figure 4:**
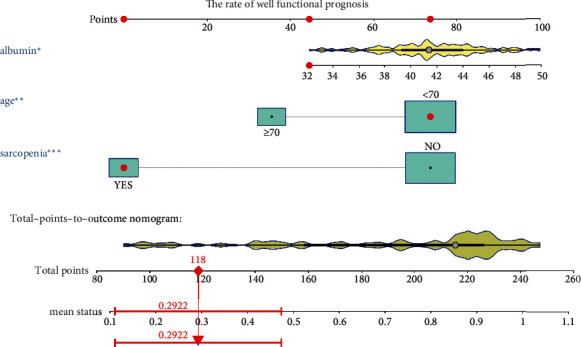
The nomogram model for predicting functional prognosis. Note: age, sarcopenia, and serum albumin levels were included. ^∗^*P* < 0.05, ^∗∗^*P* < 0.01, ^∗∗∗^*P* < 0.005.

A logistic model was then used to verify the three risk factors identified by the multifactorial linear regression model (Table 5); the obtained results indicated that age (OR 0.28 (95% CI 0.11-0.67), *P* = 0.005), sarcopenia (OR 0.11 (95% CI 0.05-0.26), *P* < 0.001), and serum albumin levels (OR 1.14 (95% CI 1.02-1.29), *P* = 0.025) were all statistically significant. Then, we used the R software to construct nomogram prediction model (Figure 4).

**Figure 5:**
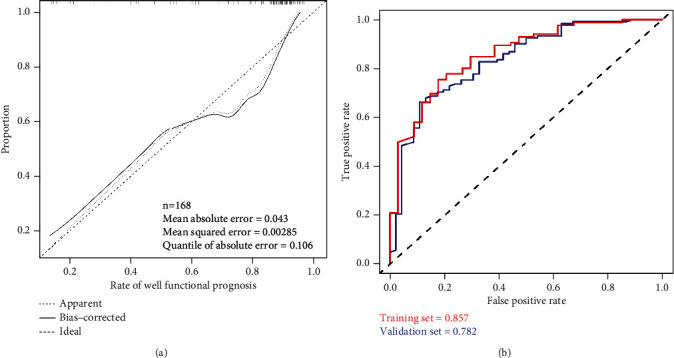
Evaluation of the nomogram prediction model. (a) A calibration curve for predicting the risk of poor functional outcome after patellar fracture in the elderly. The diagonal dashed line is the perfect prediction of the ideal model, while the solid line is the predictive power of the model. (b) The area under the curve (AUC) of a nomogram model indicates the probability of accurately predicting a poor postoperative functional outcome in a randomized patient selection scenario.

**Figure 6:**
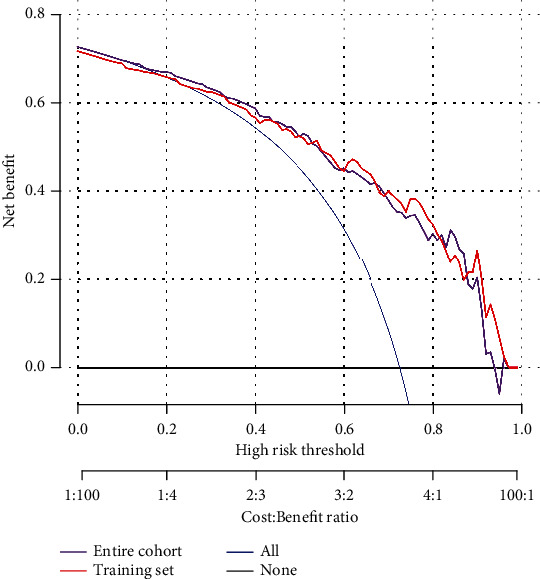
Decision analysis curve includes training test and overall groups.

The calibration curve of the nomogram was close to the perfect prediction curve of ideal model suggesting that the model can be used to predict the risk of poor functional outcome after patellar fracture surgery in the elderly patients (Figure 5(a)). The obtained AUC of this prediction model was 0.857 (95% CI (0.783-0.929)) (Figure 5(b)). In addition, the model showed a good predictive power with a C-index of 0.865 (95% CI (0.785-0.929)), 0.821 (95% CI (0.616-0.949)), and 0.836 (95% CI (0.767-0.904)) in the training set, the validation set, and the entire cohort, respectively.  Figure 6 shows the decision analysis curve of a poor prediction model for functional outcome after patella fracture in the elderly. The decision analysis curve indicated that clinical decisions based on the nomogram prediction model could better predict the recovery of functional outcome.

## 4. Discussion

The development of the R software has led to nomogram prediction models being widely used in the field of clinical prognosis assessment. Previous studies have reported that nomogram prediction models have clear quantitative indexes which can accurately evaluate the prognosis [31–33]. This retrospective study assessed, for the first time, the risk of poor functional outcome after patella fracture surgery in the elderly using a nomogram prediction model that included three variables: age, sarcopenia, and serum albumin level. The obtained results after the internal sample survey indicated that the model has a strong predictive ability. In addition, the high C-index and AUC index indicated that the predictive model can be widely and accurately used to assess the functional prognosis of patellar fractures in the elderly. The HSS score was proposed by the American Hospital for Special Surgery in 1976, and it includes six dimensions: pain, function, joint mobility, muscle strength, knee flexion deformity, and knee stability. The score has been widely used for evaluating knee functional prognosis [34]. In this study, the HSS score at 6 months postoperatively was used as an index to assess the early functional outcome of elderly patella fracture patients. In addition, multifactorial linear regression analysis, logistic regression analysis, and the R software were used to construct a nomogram. Results obtained from the nomogram indicated that advanced age, sarcopenia, and hypoalbuminemia were the main risk factors affecting the functional outcome after patella fracture surgery.

Sarcopenia was first named by Rosenberg in 1989 [35]. It is primarily characterized by loss of muscle mass, low muscle strength, and/or low physical performance [36]. Sarcopenia is a systemic muscle disease which has serious physical and clinical consequences that significantly increases medical expenses [37]. Previous studies have reported that the direct medical costs of sarcopenia in the United States in 2000 was about $18.5 billion, accounting for 1.5% of the total medical expenditures in that year [38]. The impact of sarcopenia on the functional outcome of lower extremity fractures may be more pronounced than in the upper extremity fractures because the lower limb accounts for more than 50% of the body's skeletal muscle mass [39]. This study demonstrated that sarcopenia is a risk factor for poor functional outcome after patellar fracture in the elderly. The obtained results after conducting multifactorial linear regression analysis indicated that the relative degree of regression contribution by sarcopenia was 17.3%, and its *P* value in the logistic regression was <0.001, which was consistent with the results reported in other studies [40–42]. The adverse effects of sarcopenia on functional prognosis can be attributed to the disruption of the balance between the anabolic and catabolic pathways of muscle protein metabolism, the decrease in size and number of muscle fibers, the shift from type II to type I, and the infiltration of muscle and intermuscular fat. Previous studies have reported that all these factors severely affect the normal function of the skeletal muscle in patients with sarcopenia [43–45]. On the other hand, the reduction of the skeletal muscle mass reduces the balance control of the body and the mechanical load on the skeleton, thereby resulting in less stressful bone remodeling which causes osteoporosis and low bone mass, with both bone and muscle interacting to delay postoperative functional recovery [46]. Some studies have shown that resistance training and nutritional support therapy can improve the symptoms of sarcopenia. However, the ideal index for intervention has not yet been elucidated, and thus, further research is needed [47].

This study also found that a low preoperative serum albumin level was an important risk factor for poor functional outcome after patella fracture, which is consistent with the results reported in previous studies [48–51]. Serum albumin, the main component of total serum protein, is synthesized by the liver and plays an important role in maintaining blood colloid osmolarity, in vivo transport of metabolic substances, and nutritional support. Serum albumin is regarded as an important nutritional indicator, and low serum albumin levels are directly associated with the poor nutritional status of patients. However, further studies should be conducted to determine the specific impact of nutritional interventions on functional outcomes after surgery [52, 53].

The model developed in this study can effectively predict the risk of poor functional outcome after patella fracture surgery in the elderly. This can achieve individualized risk prediction, thereby helping clinicians to identify patients having a high risk of poor functional outcome at an early stage and thus take appropriate intervention measures. However, this study had some limitations: firstly, the cohort used was not representative of all patients with patella fractures; secondly, the study did not include all possible influencing factors, although the statistical examination has validated the reliability and validity of our predictive model; and thirdly, the study did not perform tests to assess physical functions such as gait speed, short physical fitness battery (SPPB), and timed walk test (TUG) because the patella fracture limited the patient's knee motor function, which to some extent influenced the assessment of the severity of sarcopenia.

## 5. Conclusion

This study developed a predictive model for assessing the risk of poor functional outcome after patella fracture surgery in the elderly, which the model has shown to be valid and reliable. The results obtained from the model suggest that advanced age, sarcopenia, and low serum albumin levels are high risk factors for a poor functional outcome.

## Figures and Tables

**Table 1:** Demographic data of the good and bad functional prognosis groups.

	Total (*n* = 168)	Good functional prognosis (*n* = 122)	Bad functional prognosis (*n* = 46)	*P* value
Gender				
Male	63 (37.5%)	46 (37.7%)	17 (37.0%)	
Female	105 (62.5%)	76 (62.3%)	29 (63.0%)	0.929
Age (year)	66.29 ± 6.65	64.46 ± 4.90	71.15 ± 8.15	<0.001
≥60 and <70	129 (76.8%)	104 (85.2%)	25 (54.3%)	
≥70	39 (23.2%)	18 (14.8%)	21 (45.7%)	<0.001
Diabetes				
Yes	37 (22.0%)	27 (22.1%)	10 (21.7%)	
No	131 (78.0%)	95 (77.9%)	36 (78.3%)	0.956
Hypertensive disease				
Yes	50 (29.8%)	31 (25.4%)	19 (41.3%)	
No	118 (70.2%)	91 (74.6%)	27 (58.7%)	0.069
Cardiac disease				
Yes	44 (26.2%)	26 (21.3%)	18 (39.1%)	
No	124 (73.8%)	96 (78.7%)	28 (60.9%)	0.019
Hyperlipidemia				
Yes	48 (28.6%)	39 (32.0%)	9 (19.6%)	
No	120 (71.4%)	83 (68.0%)	37 (80.4%)	0.113
Sarcopenia				
Yes	43 (25.6%)	15 (12.3%)	28 (60.9%)	
No	125 (74.4%)	107 (87.7%)	18 (39.1%)	<0.001
Time to surgery (day)	2.57 ± 1.40	2.53 ± 1.43	2.67 ± 1.32	0.560
BMI (kg/m^2^)	23.06 ± 3.62	23.55 ± 3.33	20.57 ± 4.06	0.004
Albumin (g/L)	41.51 ± 3.68	42.12 ± 3.58	39.88 ± 3.49	<0.001
Hemoglobin (g/L)	132.07 ± 14.23	133.55 ± 13.91	128.14 ± 14.47	0.028
tmCSA/BW (cm^2^/kg)	1.38 ± 0.18	1.40 ± 0.18	1.33 ± 0.17	0.018
Grip strength (kg)	21.86 ± 7.48	23.09 ± 7.22	18.62 ± 7.27	<0.001
Affected side				
Right	59 (35.1%)	39 (32.0%)	20 (43.5%)	
Left	109 (64.9%)	83 (68.0%)	26 (56.5%)	0.163
Fracture type				
A	29 (17.3%)	21 (17.2%)	8 (17.4%)	
B	24 (14.2%)	14 (11.5%)	10 (21.7%)	
C	115 (68.5%)	87 (71.3%)	28 (60.9%)	0.224

The fractures are classified using the radiological assessment method of the AO classification system. ^∗^Statistically significant difference (*P* < 0.05).

**Table 2:** Radiologic outcomes at six months postoperatively.

Radiologic outcomes	Good prognosis	Bad prognosis	*P* value
Insall-Salvati index	0.99 ± 0.15	1.02 ± 0.14	0.141
Blackburne-Peel index	0.92 ± 0.16	0.89 ± 0.18	0.272
Congruence angle (degree)	1.80 ± 4.32	2.32 ± 4.09	0.483
Lateral patellofemoral angle (degree)	7.23 ± 2.66	6.78 ± 2.69	0.327

^∗^Statistically significant difference (*P* < 0.05).

**Table 3:** Functional outcomes after surgery for patellar fracture.

Functional outcomes		Improved group	Unimproved group	*P* value
Range of motion (%)	Month 3	78.33 ± 4.41	77.15 ± 5.47	0.150
	Month 6	84.74 ± 4.55	83.82 ± 5.16	0.259
HSS scores (%)	Month 3	81.49 ± 6.22	68.87 ± 5.43	<0.001
	Month 6	88.48 ± 5.31	74.41 ± 5.08	<0.001

^∗^Statistically significant difference (*P* < 0.05).

**Table 4:** Multivariable regression analyses for independent predictors of HSS knee scores at 6 months postoperatively.

Model	Predictors	Beta	*P* value	Partial *R*^2^	Tolerance	VIF
Multivariable	Age	-0.48	<0.001	0.405	0.889	1.125
	Sarcopenia	-0.42	<0.001	0.173	0.876	1.141
	Albumin	0.14	0.007	0.016	0.938	1.066

Linear regression analysis and forward stepwise variable selection with residual analysis of variance were used to determine if the model had linear trend, independence, normality, and variance congruence. VIF: variance inflation factor.

**Table 5:** Chart of prediction factors.

Variable	Prediction model		
	*β*	Odds ratio (95% CI)	*P* value
(Intercept)	-3.442	0.03 (0-4.06)	0.163
Sarcopenia	-2.173	0.11 (0.05-0.26)	<0.001
Age	-1.287	0.28 (0.11-0.67)	0.005
Albumin	0.134	1.14 (1.02-1.29)	0.025

*β* is the regression coefficient.

## Data Availability

We provided the original data of this manuscript in the supplementary information files.

## Supplementary Materials

Supplementary Materials This supplemental file is the original data for the 168 patients enrolled in the manuscript during preoperative and postoperative follow-up, which contains patient ID, functional outcome, sarcopenia, age, affect side, cardiac disease, hypertensive disease, diabetes, hyperlipidemia, BMI, grip strength, tmCSA/BW, albumin, hemoglobin, AO type, wait time for surgery, etc.
