# Supplementary material for: A Prediction Modeling Based on the Hospital for Special Surgery (HSS) Knee Score for Poor Postoperative Functional Prognosis of Elderly Patients with Patellar Fractures
Source: Biomed Res Int. 2021 Dec 6;2021:6620504. doi: 10.1155/2021/6620504 (PMC8668305; doi:10.1155/2021/6620504)
Supplement: Supplementary Materials — This supplemental file is the original data for the 168 patients enrolled in the manuscript during preoperative and postoperative follow-up, which contains patient ID, functional outcome, sarcopenia, age, affect side, cardiac disease, hypertensive disease, diabetes, hyperlipidemia, BMI, grip strength, tmCSA/BW, albumin, hemoglobin, AO type, wait time for surgery, etc. [file 6620504.f1.pdf]

Original data of the paper

This supplemental file is the original data for the 168 patients enrolled in the manuscript during preoperative and postoperative follow-up，which contains patient ID,functional outcome ,sarcopenia,age,affect side,cardiac disease,hypertensive disease,diabetes,hyperlipidemia,bmi,grip strength,tmCSA/ BW,albumin,hemoglobin,AO type,wait time for surgery,etal.

R3,R6(Knee range of motion at 3,6 months postoperatively)

HSS3,HSS6(HSS score at 3,6 months postoperatively)

| Number | Patient ID | Functiona l outcome | Sarcopenia | Age | ≥ 70y | Sex | Affect side | Cardiac disease | Hypertensiv e disease | Diabetes | Hyperli-pidemia | Bmi | Grip strengt h | tmCSA/ BW | Albumi n | Hemoglobi n | Wait time for surger | AO | R3 | R6 | HSS 3 | HSS 6 | IS | BP | CA | LFA |
|---|---|---|---|---|---|---|---|---|---|---|---|---|---|---|---|---|---|---|---|---|---|---|---|---|---|---|
| 1 | 1250859 | bad | yes | 81 | yes | male | left | no | yes | no | no | 17.21 | 20.4 | 1.27 | 41 | 134.6 | 4 | 3 | 83.38 | 92.83 | 64 | 68 | 1.14 | 0.74 | -5.15 | 6.73 |
| 2 | 1280273 | good | no | 63 | no | male | right | yes | no | yes | no | 23.31 | 25.8 | 1.6 | 42.7 | 154 | 1 | 3 | 73.24 | 79.79 | 83 | 92 | 0.97 | 1.11 | 5.03 | 4.88 |
| 3 | 1290430 | bad | yes | 84 | yes | female | right | no | yes | yes | no | 19.53 | 10.5 | 1.22 | 38.4 | 105 | 3 | 1 | 77.59 | 85.38 | 59 | 65 | 1.15 | 0.66 | -2.28 | 8.32 |
| 4 | 1290491 | good | no | 63 | no | female | left | no | no | no | yes | 22.04 | 23.4 | 1.42 | 45.5 | 135 | 1 | 2 | 83.79 | 88.07 | 80 | 86 | 0.96 | 0.93 | 3.98 | 5.06 |
| 5 | 1291468 | good | no | 61 | no | female | right | no | no | yes | no | 32.79 | 21.2 | 1.18 | 40.2 | 126 | 4 | 3 | 79.24 | 82.41 | 85 | 94 | 0.9 | 0.94 | -3.55 | 7.38 |
| 6 | 1298717 | bad | yes | 74 | yes | female | left | no | no | no | no | 20.81 | 13.7 | 1.24 | 37.9 | 107 | 2 | 2 | 77.03 | 86.14 | 71 | 78 | 1.48 | 1.05 | 4.05 | 8.45 |
| 7 | 1309677 | bad | yes | 90 | yes | male | right | yes | yes | no | no | 23.44 | 11.2 | 1.23 | 35.4 | 117 | 4 | 3 | 73.03 | 81.52 | 58 | 66 | 1 | 0.87 | 7.31 | 12.83 |
| 8 | 1310910 | good | no | 62 | no | male | right | yes | yes | no | yes | 37.11 | 34.6 | 1.6 | 43 | 148 | 5 | 3 | 70.55 | 79.59 | 79 | 86 | 0.87 | 0.86 | -5.08 | 9.48 |
| 9 | 1310912 | good | no | 63 | no | female | right | no | no | no | yes | 20.83 | 22.3 | 1.26 | 44.3 | 127 | 5 | 3 | 83.03 | 88.9 | 81 | 87 | 0.97 | 0.93 | -4.39 | 9.27 |
| 10 | 1319040 | bad | yes | 69 | no | female | right | no | no | no | yes | 18.73 | 12.3 | 1.23 | 45.1 | 138 | 5 | 1 | 85.79 | 90.07 | 72 | 74 | 1.01 | 0.81 | 6.79 | 5.26 |
| 11 | 1325781 | good | no | 78 | yes | female | right | no | yes | no | no | 22.83 | 15.8 | 1.32 | 38.3 | 111 | 3 | 3 | 74.69 | 83.66 | 74 | 81 | 0.97 | 0.83 | -3.43 | 14.71 |
| 12 | 1329822 | bad | no | 69 | no | male | left | no | yes | no | no | 21.22 | 25.2 | 1.61 | 32.9 | 135 | 1 | 3 | 77.79 | 83.79 | 72 | 77 | 0.98 | 0.83 | 6.02 | 6.93 |
| 13 | 1338560 | good | no | 60 | no | female | left | no | no | no | no | 23.88 | 25.8 | 1.28 | 45.7 | 121 | 4 | 3 | 80.48 | 84.41 | 87 | 97 | 0.75 | 0.99 | 6.69 | 2.15 |
| 14 | 1340350 | good | no | 60 | no | female | right | no | yes | no | no | 25.72 | 23.4 | 1.34 | 41.1 | 129 | 1 | 3 | 82.97 | 89.38 | 88 | 96 | 0.84 | 0.76 | -6.73 | 10.46 |
| 15 | 1343665 | good | no | 77 | yes | female | left | no | yes | no | no | 22.03 | 17.2 | 1.32 | 44 | 119 | 4 | 3 | 74.97 | 82.62 | 75 | 81 | 1.22 | 0.9 | -5.65 | 7.12 |
| 16 | 1349370 | bad | yes | 64 | no | female | left | yes | no | no | no | 20.58 | 15.9 | 1.21 | 41 | 122 | 2 | 3 | 79.45 | 88.76 | 76 | 78 | 0.93 | 0.86 | 1.6 | 2.65 |
| 17 | 1349859 | good | no | 65 | no | female | left | no | no | yes | no | 24.89 | 22.7 | 1.25 | 39 | 118 | 5 | 3 | 81.52 | 87.72 | 83 | 85 | 0.99 | 0.81 | 5.14 | 4.76 |
| 18 | 1351467 | good | no | 69 | no | female | left | no | no | yes | no | 20.4 | 19.5 | 1.6 | 44 | 110 | 2 | 3 | 82.41 | 86.28 | 77 | 85 | 0.73 | 0.58 | 5.48 | 9.24 |
| 19 | 1351286 | good | no | 76 | yes | female | left | no | no | no | no | 20.8 | 19.8 | 1.22 | 45.5 | 135 | 1 | 3 | 75.31 | 82 | 68 | 81 | 1.11 | 1.08 | 6.68 | 5.26 |
| 20 | 1352667 | good | no | 62 | no | male | left | yes | yes | no | no | 23.14 | 36.4 | 1.72 | 41.7 | 144 | 3 | 2 | 80.28 | 86.07 | 80 | 90 | 0.96 | 0.85 | -4.56 | 6.12 |
| 21 | 1354821 | good | no | 63 | no | female | left | no | no | no | no | 28.13 | 21.5 | 1.28 | 46.1 | 136 | 2 | 3 | 69.72 | 76.07 | 82 | 92 | 0.81 | 0.76 | 8.04 | 8.11 |
| 22 | 1358306 | bad | no | 68 | no | male | right | no | yes | no | no | 22.49 | 35.6 | 1.35 | 40.5 | 146 | 3 | 3 | 75.72 | 82.9 | 72 | 76 | 1.05 | 0.67 | -2.12 | 3.18 |
| 23 | 1359575 | good | no | 61 | no | female | left | yes | no | no | yes | 25.4 | 22.9 | 1.32 | 47 | 151 | 3 | 3 | 77.59 | 83.93 | 83 | 89 | 0.72 | 1.09 | 4.58 | 6.63 |
| 24 | 1359704 | good | no | 62 | no | male | right | no | no | yes | yes | 27.68 | 37.3 | 1.4 | 42.2 | 158 | 3 | 3 | 82.21 | 89.86 | 79 | 86 | 0.76 | 0.78 | 3.39 | 3.45 |
| 25 | 1363889 | good | no | 62 | no | female | left | no | no | no | no | 21.48 | 23.2 | 1.32 | 44.7 | 150 | 3 | 1 | 83.17 | 90.48 | 71 | 81 | 1.02 | 0.95 | 6.67 | 4.12 |
| 26 | 1365687 | good | no | 70 | yes | female | left | yes | no | no | no | 24.03 | 18.4 | 1.32 | 41.1 | 110 | 3 | 3 | 75.31 | 79.59 | 76 | 83 | 0.84 | 0.79 | -0.98 | 5.64 |
| 27 | 1368393 | good | no | 61 | no | female | right | no | no | yes | yes | 23.44 | 20.3 | 1.38 | 43.6 | 152 | 3 | 3 | 79.52 | 86.83 | 80 | 86 | 1.02 | 0.99 | 4.34 | 3.08 |
| 28 | 1370529 | good | no | 62 | no | female | left | yes | no | no | yes | 24.22 | 17.8 | 1.26 | 49.8 | 115 | 5 | 3 | 80.48 | 86.07 | 80 | 90 | 0.96 | 0.67 | -4.82 | 6.34 |
| 29 | 1375080 | bad | yes | 85 | yes | female | left | no | yes | yes | yes | 16.65 | 12.4 | 1.25 | 44.2 | 121 | 3 | 3 | 87.31 | 90.62 | 62 | 68 | 1.03 | 0.83 | 5.88 | 8.26 |
| 30 | 1377364 | good | yes | 64 | no | male | right | yes | no | no | no | 20.76 | 20.6 | 1.4 | 40.6 | 151 | 4 | 3 | 77.59 | 85.59 | 83 | 86 | 1.13 | 1.05 | 2.82 | 7.86 |
| 31 | 1377411 | bad | yes | 67 | no | female | left | yes | no | yes | no | 19.53 | 15.1 | 1.23 | 40.2 | 122 | 3 | 3 | 74.28 | 83.1 | 71 | 76 | 0.98 | 0.82 | 3.05 | 4.18 |
| 32 | 1378576 | good | no | 61 | no | female | left | yes | yes | yes | no | 24.84 | 23.5 | 1.32 | 40.9 | 125 | 2 | 1 | 77.38 | 81.03 | 85 | 93 | 0.81 | 1.11 | 4.3 | 6.94 |
| 33 | 1378636 | good | yes | 60 | no | female | left | no | no | no | yes | 17.58 | 13.7 | 1.25 | 43 | 116 | 4 | 1 | 82.62 | 88.48 | 79 | 82 | 0.8 | 0.85 | -3.93 | 5.29 |
| 34 | 1378666 | good | no | 61 | no | female | left | no | no | no | no | 24.65 | 21.4 | 1.46 | 43.3 | 124 | 3 | 1 | 82 | 86.97 | 85 | 91 | 0.95 | 0.68 | 5.02 | 5.93 |
| 35 | 1380991 | good | no | 60 | no | male | right | yes | no | no | no | 24.93 | 34.5 | 1.59 | 39.7 | 151 | 1 | 3 | 80.76 | 90.55 | 89 | 94 | 0.98 | 0.88 | -3.06 | 7.64 |
| 36 | 1381811 | bad | no | 63 | no | female | left | no | no | no | no | 19.05 | 22.3 | 1.35 | 47.9 | 137 | 1 | 3 | 84.69 | 89.66 | 72 | 76 | 0.93 | 0.76 | 3.48 | 9.16 |
| 37 | 1386510 | good | no | 61 | no | female | right | no | no | no | yes | 26.18 | 19.1 | 1.28 | 43.7 | 148 | 2 | 3 | 73.31 | 78.34 | 85 | 94 | 1.2 | 0.84 | 2.33 | 6.97 |
| 38 | 1389678 | good | yes | 60 | no | female | right | no | no | no | no | 19.53 | 17.2 | 1.21 | 44.3 | 128 | 5 | 3 | 77.38 | 82.62 | 82 | 85 | 0.77 | 1.22 | 4.16 | 9.85 |
| 39 | 1392523 | good | no | 63 | no | male | left | no | no | yes | no | 31.35 | 30.4 | 1.37 | 47.6 | 158 | 4 | 3 | 74.62 | 79.86 | 83 | 96 | 1.2 | 0.97 | 0.22 | 10.12 |
| 40 | 1394612 | good | no | 64 | no | male | left | no | no | no | no | 25.39 | 33.9 | 1.6 | 43.2 | 135 | 4 | 3 | 74.28 | 82.21 | 76 | 85 | 1.23 | 0.82 | 4.13 | 3.49 |
| 41 | 1397633 | good | no | 60 | no | male | left | no | no | yes | no | 26.3 | 36.7 | 1.36 | 43.4 | 134 | 4 | 2 | 72.41 | 76.9 | 88 | 96 | 1.35 | 0.78 | -2.99 | 4.75 |

| Number | Patient ID | Functional outcome | Sarcopenia | Age | ⩾ 70y | Sex | Affect side | Cardiac disease | Hypertensive disease | Diabetes | Hyperli-pidemia | Bmi | Grip strength | tmCSA/BW | Albumin | Hemoglobin | Wait time for surger | AO | R3 | R6 | HSS 3 | HSS 6 | IS | BP | CA | LFA |
|---|---|---|---|---|---|---|---|---|---|---|---|---|---|---|---|---|---|---|---|---|---|---|---|---|---|---|
| 42 | 1398175 | good | no | 76 | yes | female | left | yes | no | no | no | 24.44 | 15.3 | 1.28 | 42.6 | 138 | 1 | 1 | 76.62 | 83.38 | 75 | 84 | 0.91 | 0.92 | 6.66 | 9.18 |
| 43 | 1401149 | bad | yes | 70 | yes | female | left | no | no | no | no | 20 | 10.3 | 1.22 | 46.5 | 129 | 4 | 3 | 68.14 | 77.17 | 61 | 67 | 0.84 | 0.77 | -3.35 | 5.64 |
| 44 | 1399585 | good | no | 71 | yes | female | right | yes | yes | no | no | 28 | 18.6 | 1.37 | 41.7 | 135 | 1 | 3 | 72.07 | 79.79 | 77 | 82 | 1.1 | 0.9 | 6.66 | 10.28 |
| 45 | 1401346 | good | no | 61 | no | female | left | no | no | no | yes | 26.93 | 18.5 | 1.26 | 43.2 | 148 | 1 | 3 | 73.38 | 77.59 | 80 | 90 | 0.75 | 0.87 | 0.15 | 8.42 |
| 46 | 1402769 | bad | no | 65 | no | female | left | no | no | no | no | 18.9 | 16.2 | 1.49 | 41.3 | 117 | 2 | 1 | 82.83 | 92.21 | 70 | 80 | 1.23 | 0.71 | 4.9 | 5.36 |
| 47 | 1403349 | good | no | 63 | no | female | left | no | no | no | no | 19.95 | 19.7 | 1.24 | 42.7 | 126 | 2 | 3 | 81.72 | 93.03 | 74 | 83 | 1.13 | 0.78 | -1.99 | 9.98 |
| 48 | 1404488 | good | no | 63 | no | female | left | no | no | no | no | 23.88 | 22.4 | 1.35 | 47.6 | 146 | 4 | 3 | 80.14 | 83.66 | 84 | 90 | 0.92 | 0.77 | 3.86 | 6.48 |
| 49 | 1405480 | good | no | 76 | yes | female | left | no | no | yes | no | 21.48 | 13.3 | 1.4 | 44.9 | 132 | 2 | 3 | 76.97 | 81.66 | 76 | 83 | 1.02 | 0.9 | 5.59 | 7.45 |
| 50 | 1408808 | bad | yes | 64 | no | female | left | yes | yes | no | yes | 24.86 | 13.2 | 1.11 | 45.4 | 153 | 6 | 2 | 69.17 | 74.76 | 75 | 80 | 1.25 | 0.71 | 2.36 | 5.62 |
| 51 | 1411126 | bad | yes | 65 | no | female | right | no | no | no | no | 18.07 | 17.1 | 1.24 | 40.8 | 117 | 1 | 2 | 79.66 | 83.17 | 73 | 77 | 0.93 | 1.09 | -1.83 | 9.82 |
| 52 | 1415155 | good | yes | 62 | no | female | left | no | no | no | no | 20.4 | 12.5 | 1.24 | 42.4 | 135 | 2 | 2 | 77.72 | 82.21 | 80 | 83 | 0.91 | 0.71 | 4.27 | 2.34 |
| 53 | 1420645 | good | no | 62 | no | female | left | yes | no | yes | yes | 24.03 | 15.1 | 1.44 | 41.4 | 117 | 2 | 1 | 77.66 | 84.97 | 81 | 90 | 0.79 | 1.47 | -0.04 | 8.24 |
| 54 | 1422159 | good | no | 67 | no | male | right | yes | no | no | no | 21.22 | 24.3 | 1.68 | 43.7 | 146 | 5 | 3 | 71.93 | 77.79 | 71 | 81 | 0.87 | 0.75 | 4.07 | 8.65 |
| 55 | 1422163 | good | yes | 60 | no | female | left | no | no | no | no | 30.82 | 13.6 | 1.07 | 39.2 | 135 | 2 | 2 | 72.28 | 78.69 | 78 | 83 | 1.17 | 0.56 | 3.56 | 6.45 |
| 56 | 1425769 | good | no | 68 | no | female | left | no | no | no | no | 20.96 | 16.3 | 1.35 | 40.3 | 117 | 2 | 3 | 77.24 | 84.48 | 73 | 83 | 0.88 | 1.06 | 3.57 | 4.32 |
| 57 | 1428133 | good | no | 60 | no | male | left | no | no | yes | yes | 24.49 | 36.7 | 1.49 | 42.8 | 132 | 4 | 3 | 70.69 | 76.83 | 87 | 93 | 0.85 | 0.67 | 5.06 | 11.45 |
| 58 | 1428952 | bad | yes | 63 | no | male | right | yes | no | no | no | 22.84 | 22.8 | 1.24 | 39.3 | 156 | 4 | 3 | 77.03 | 81.1 | 73 | 79 | 0.8 | 1.54 | 5.11 | 4.75 |
| 59 | 1429359 | good | no | 63 | no | male | left | no | no | no | yes | 26.99 | 33.3 | 1.59 | 49.3 | 140 | 1 | 3 | 78.07 | 84.97 | 82 | 92 | 1.15 | 0.72 | -4.74 | 10.37 |
| 60 | 1429837 | good | no | 63 | no | female | left | yes | no | no | yes | 25.4 | 20.5 | 1.28 | 47 | 151 | 3 | 3 | 79.79 | 87.45 | 94 | 96 | 0.84 | 1.32 | 3.76 | 6.4 |
| 61 | 1431246 | good | no | 61 | no | male | right | no | no | yes | yes | 31.14 | 27.4 | 1.28 | 42.2 | 158 | 3 | 2 | 77.86 | 83.79 | 92 | 96 | 0.8 | 1.02 | 3.32 | 12.76 |
| 62 | 1432170 | good | no | 64 | no | female | left | no | no | yes | yes | 23.24 | 16.8 | 1.37 | 41.1 | 126 | 1 | 3 | 81.66 | 89.38 | 86 | 95 | 0.91 | 1.09 | -3.13 | 7.26 |
| 63 | 1432586 | good | no | 63 | no | male | left | no | no | no | no | 26.83 | 35.2 | 1.62 | 44 | 133 | 4 | 1 | 78.76 | 88.34 | 80 | 86 | 0.98 | 0.96 | -3.82 | 6.54 |
| 64 | 1434082 | good | no | 61 | no | female | left | no | no | no | no | 19.53 | 15.7 | 1.44 | 42.2 | 135 | 4 | 2 | 82.41 | 90.97 | 74 | 84 | 0.88 | 0.75 | 4.7 | 8.16 |
| 65 | 1434359 | good | no | 62 | no | female | right | no | no | no | no | 22.04 | 20.3 | 1.29 | 48.3 | 124 | 2 | 3 | 76.28 | 85.1 | 81 | 93 | 1.02 | 0.99 | 4.61 | 7.73 |
| 66 | 1434764 | bad | no | 60 | no | male | left | yes | no | no | no | 29.32 | 38.5 | 1.6 | 44.2 | 147 | 2 | 3 | 72.48 | 80.21 | 70 | 80 | 0.96 | 0.9 | -4.06 | 5.12 |
| 67 | 1436578 | good | no | 66 | no | female | left | yes | no | no | no | 23.66 | 18.1 | 1.22 | 47.2 | 132 | 1 | 3 | 77.72 | 85.93 | 84 | 93 | 0.97 | 1.06 | 5.74 | 3.46 |
| 68 | 1436758 | bad | yes | 68 | no | female | left | yes | no | no | no | 19.53 | 16.4 | 1.21 | 41.8 | 133 | 5 | 3 | 78.83 | 87.38 | 61 | 67 | 0.98 | 0.93 | 1.37 | 8.2 |
| 69 | 1437148 | bad | yes | 80 | yes | female | right | no | yes | no | no | 24.82 | 15.1 | 1.12 | 41 | 110 | 3 | 3 | 70.48 | 76.69 | 75 | 79 | 1.07 | 0.82 | -5.93 | 7.41 |
| 70 | 1437458 | good | no | 63 | no | male | right | no | no | no | yes | 27.92 | 33.8 | 1.19 | 39.4 | 138 | 1 | 3 | 71.38 | 78.69 | 88 | 95 | 0.71 | 1.16 | -1.94 | 3.59 |
| 71 | 1438979 | good | no | 67 | no | male | left | no | no | no | yes | 24.51 | 24.3 | 1.21 | 41.3 | 152 | 3 | 3 | 76.48 | 83.24 | 76 | 83 | 1.02 | 0.76 | 2.5 | 5.1 |
| 72 | 1439505 | good | no | 68 | no | male | left | no | no | no | no | 23.15 | 25.9 | 1.33 | 43.1 | 129 | 6 | 3 | 77.52 | 86.62 | 75 | 82 | 0.72 | 1.12 | 5.72 | 4.07 |
| 73 | 1440078 | bad | yes | 86 | yes | female | left | no | no | no | yes | 16.44 | 13.2 | 1.25 | 41.3 | 119 | 2 | 3 | 75.79 | 82 | 56 | 63 | 0.98 | 0.75 | 1.83 | 5.35 |
| 74 | 1440252 | bad | no | 67 | no | female | left | no | yes | yes | no | 39.45 | 19.6 | 1.05 | 41.3 | 124 | 3 | 1 | 73.45 | 78.07 | 77 | 80 | 1.11 | 1.15 | -0.02 | 3.75 |
| 75 | 1442294 | bad | yes | 79 | yes | female | right | yes | no | yes | yes | 22.22 | 14.7 | 1.23 | 39.2 | 104 | 1 | 1 | 76.28 | 82.9 | 60 | 68 | 1.13 | 0.96 | 4.26 | 5.26 |
| 76 | 1447291 | good | no | 61 | no | male | right | yes | yes | no | no | 19.66 | 35.8 | 1.67 | 39.7 | 151 | 1 | 3 | 83.24 | 90.34 | 72 | 82 | 1.06 | 0.83 | -1.15 | 7.32 |
| 77 | 1447375 | good | no | 61 | no | male | left | no | yes | no | no | 23.89 | 37.2 | 1.6 | 42.4 | 139 | 3 | 3 | 74.34 | 83.72 | 90 | 94 | 0.88 | 0.85 | -2.37 | 8.55 |
| 78 | 1447622 | good | yes | 70 | yes | male | left | no | no | no | no | 24.49 | 21.3 | 1.25 | 37.9 | 158 | 2 | 3 | 75.38 | 80.9 | 78 | 84 | 0.91 | 0.77 | 2.95 | 11.37 |
| 79 | 1447612 | good | no | 69 | no | female | left | no | yes | yes | no | 22.03 | 14.5 | 1.22 | 41.1 | 119 | 2 | 3 | 66.48 | 72.62 | 76 | 81 | 1.07 | 0.92 | 5 | 6.58 |
| 80 | 1447732 | good | no | 60 | no | female | right | no | no | yes | no | 21.48 | 21.1 | 1.48 | 46.3 | 142 | 5 | 3 | 82.62 | 88.62 | 93 | 96 | 1.56 | 1.4 | -3.82 | 6.32 |
| 81 | 1453374 | bad | no | 64 | no | male | right | yes | yes | no | no | 16.65 | 31.4 | 1.56 | 37.5 | 104 | 2 | 1 | 82.97 | 90.28 | 68 | 74 | 1.29 | 1.11 | 2.72 | 2.66 |
| 82 | 1453821 | good | no | 61 | no | female | left | no | no | no | no | 21.19 | 19.5 | 1.32 | 46 | 127 | 1 | 3 | 77.66 | 83.93 | 89 | 96 | 1.02 | 1.02 | -3.01 | 8.08 |
| 83 | 1454506 | good | no | 60 | no | female | left | no | yes | no | yes | 19.78 | 20.9 | 1.45 | 49.9 | 144 | 1 | 1 | 84.21 | 90.76 | 75 | 86 | 0.85 | 1.03 | -1.43 | 3.25 |
| 84 | 1455242 | good | no | 72 | yes | female | left | no | no | yes | no | 29.69 | 16.7 | 1.04 | 45.1 | 124 | 4 | 3 | 78.28 | 81.52 | 75 | 87 | 0.84 | 0.93 | 4.35 | 12.37 |
| 85 | 1456366 | good | yes | 60 | no | female | right | no | no | no | no | 17.97 | 11.6 | 1.25 | 41.5 | 122 | 2 | 3 | 87.03 | 91.66 | 81 | 88 | 1.02 | 1.06 | 4.49 | 8.48 |
| 86 | 1456992 | bad | yes | 79 | yes | male | right | no | no | no | no | 18.29 | 18.7 | 1.5 | 37 | 129 | 5 | 3 | 75.66 | 84.41 | 71 | 72 | 1.17 | 0.89 | 5.41 | 3.28 |
| 87 | 1458449 | bad | yes | 66 | no | female | left | yes | yes | yes | no | 19.92 | 13.3 | 1.24 | 39.3 | 130 | 1 | 1 | 85.03 | 89.38 | 75 | 78 | 1.1 | 0.7 | 3.92 | 8.42 |
| 88 | 1461896 | good | no | 69 | no | male | left | no | no | no | yes | 24.49 | 31 | 1.15 | 43.7 | 144 | 6 | 3 | 79.17 | 83.1 | 78 | 84 | 1.06 | 0.99 | -2.93 | 6.54 |

| Number | Patient ID | Functional outcome | Sarcopenia | Age | ≥ 70y | Sex | Affect side | Cardiac disease | Hypertensive disease | Diabetes | Hyperli-pidemia | Bmi | Grip strength | tmCSA/BW | Albumin | Hemoglobin | Wait time for surger | AO | R3 | R6 | HSS 3 | HSS 6 | IS | BP | CA | LFA |
|---|---|---|---|---|---|---|---|---|---|---|---|---|---|---|---|---|---|---|---|---|---|---|---|---|---|---|
| 89 | 1462223 | good | no | 68 | no | female | left | no | yes | no | no | 25.61 | 14.9 | 1.28 | 40.6 | 147 | 1 | 1 | 74.83 | 81.86 | 72 | 82 | 0.91 | 0.91 | 5.19 | 6.27 |
| 90 | 1464275 | good | no | 82 | yes | female | left | no | no | yes | no | 22.55 | 18.4 | 1.46 | 42.4 | 137 | 6 | 3 | 72.07 | 80.97 | 82 | 91 | 1.14 | 0.75 | 4.41 | 6.81 |
| 91 | 1466065 | good | no | 61 | no | female | right | no | no | no | no | 25.71 | 20.1 | 1.28 | 39.1 | 108 | 4 | 1 | 73.93 | 80.21 | 74 | 83 | 1.12 | 0.99 | 2.42 | 5.2 |
| 92 | 1467739 | good | yes | 61 | no | female | left | yes | no | no | no | 20.2 | 13.5 | 1.23 | 41.2 | 137 | 2 | 3 | 73.52 | 82.41 | 80 | 83 | 1.04 | 1.08 | 2.15 | 3.46 |
| 93 | 1471112 | good | no | 60 | no | male | left | no | no | yes | yes | 20.52 | 29.3 | 1.7 | 41.5 | 133 | 1 | 3 | 86.41 | 91.38 | 86 | 94 | 0.98 | 0.88 | 5.62 | 8.55 |
| 94 | 1473652 | bad | yes | 69 | no | female | right | no | no | no | no | 16.44 | 11.8 | 1.25 | 35 | 120 | 1 | 3 | 76.07 | 82.83 | 70 | 76 | 0.91 | 0.9 | -3.29 | 6.25 |
| 95 | 1474880 | bad | no | 63 | no | male | left | no | no | no | no | 24.22 | 34.6 | 1.65 | 36.7 | 144 | 3 | 3 | 77.79 | 84.97 | 69 | 76 | 1.14 | 0.91 | 4.96 | 5.31 |
| 96 | 1476317 | bad | no | 76 | yes | female | right | no | no | no | yes | 21.5 | 17.1 | 1.22 | 37.9 | 137 | 4 | 2 | 70.83 | 77.17 | 68 | 79 | 1.12 | 1.18 | 5.05 | 7.2 |
| 97 | 1476802 | good | no | 63 | no | female | right | yes | no | no | yes | 27.01 | 20.2 | 1.27 | 40 | 129 | 3 | 1 | 77.31 | 84.62 | 84 | 92 | 1.04 | 0.93 | 5.51 | 7.85 |
| 98 | 1479952 | good | no | 69 | no | female | left | no | no | yes | yes | 28 | 15.2 | 1.59 | 41.3 | 140 | 1 | 3 | 76.83 | 83.79 | 83 | 91 | 1.22 | 0.75 | -4.02 | 9.46 |
| 99 | 1483943 | good | no | 68 | no | female | right | no | yes | no | no | 22.27 | 21.1 | 1.24 | 41.4 | 125 | 2 | 1 | 71.17 | 78.41 | 80 | 87 | 0.95 | 1.51 | 5.59 | 5.36 |
| 100 | 1487936 | good | no | 68 | no | male | right | no | yes | no | no | 21.72 | 29.1 | 1.74 | 45.5 | 149 | 1 | 3 | 72.55 | 77.45 | 85 | 92 | 0.83 | 0.93 | -3.93 | 6.29 |
| 101 | 1488462 | bad | no | 61 | no | male | left | no | no | yes | no | 24.98 | 33.2 | 1.61 | 37.6 | 148 | 2 | 2 | 78.83 | 83.03 | 70 | 79 | 1.03 | 0.71 | 5.91 | 8.32 |
| 102 | 1488539 | good | no | 60 | no | male | left | no | no | no | no | 21.08 | 36.9 | 1.76 | 42.7 | 147 | 2 | 3 | 81.1 | 88.69 | 81 | 94 | 0.96 | 0.88 | -1.13 | 3.54 |
| 103 | 1489862 | bad | no | 70 | yes | female | left | no | no | no | yes | 21.63 | 17.3 | 1.45 | 45.2 | 130 | 3 | 3 | 77.59 | 81.45 | 75 | 79 | 0.9 | 0.76 | 6.57 | 6.87 |
| 104 | 1491003 | bad | yes | 71 | yes | male | right | yes | no | yes | no | 23.05 | 18.1 | 1.29 | 41.7 | 144 | 5 | 3 | 74.69 | 81.86 | 72 | 80 | 0.9 | 1.14 | 2.88 | 5.3 |
| 105 | 1495615 | bad | yes | 72 | yes | female | right | no | yes | yes | no | 23.11 | 11.3 | 1.21 | 37.6 | 122 | 2 | 3 | 67.93 | 75.59 | 65 | 67 | 1.06 | 0.87 | 5.21 | 6.75 |
| 106 | 1496435 | good | no | 62 | no | female | left | no | no | no | no | 21.83 | 19.5 | 1.23 | 47.1 | 133 | 1 | 3 | 84.07 | 92.9 | 91 | 96 | 1.12 | 0.96 | -4.46 | 4.34 |
| 107 | 1497785 | good | no | 61 | no | female | left | no | no | no | yes | 23.31 | 25 | 1.31 | 48.3 | 138 | 1 | 3 | 79.86 | 85.03 | 93 | 98 | 0.85 | 0.96 | 4.67 | 5.26 |
| 108 | 1498745 | bad | no | 60 | no | female | left | yes | no | no | no | 20.2 | 20.9 | 1.63 | 33.9 | 114 | 3 | 3 | 82.28 | 90.69 | 68 | 76 | 1.08 | 0.81 | 5.18 | 2.98 |
| 109 | 1499170 | good | yes | 65 | no | male | left | no | no | no | no | 18.59 | 23.4 | 1.57 | 35.1 | 145 | 1 | 3 | 82.28 | 88.55 | 79 | 85 | 1.1 | 0.97 | 7.13 | 3.76 |
| 110 | 1502285 | good | no | 61 | no | female | left | no | no | no | yes | 23.42 | 20.1 | 1.36 | 44.6 | 129 | 2 | 3 | 78.76 | 86.07 | 89 | 92 | 0.78 | 1.18 | -3.13 | 4.26 |
| 111 | 1502191 | good | no | 61 | no | female | left | no | no | no | no | 20.31 | 18.7 | 1.22 | 37.6 | 131 | 2 | 3 | 84.69 | 94.21 | 86 | 94 | 1.02 | 0.73 | 5.65 | 7.11 |
| 112 | 1503506 | good | no | 64 | no | female | left | yes | no | no | no | 24.16 | 13.4 | 1.27 | 44.3 | 129 | 1 | 1 | 77.86 | 82.34 | 83 | 92 | 0.98 | 0.81 | 3.49 | 8.27 |
| 113 | 1503858 | bad | no | 67 | no | male | right | no | yes | no | no | 23.36 | 25.9 | 1.61 | 35.8 | 155 | 1 | 3 | 76.21 | 84.48 | 65 | 75 | 1.12 | 0.86 | 6.27 | 4.4 |
| 114 | 1504705 | bad | yes | 77 | yes | female | left | yes | no | no | no | 19.92 | 13.8 | 1.22 | 35.6 | 114 | 3 | 1 | 80.97 | 84.97 | 66 | 72 | 1.08 | 0.79 | -5.31 | 5.43 |
| 115 | 1505007 | good | no | 73 | yes | female | left | yes | yes | no | no | 20.28 | 16.6 | 1.37 | 49.1 | 138 | 1 | 2 | 76.97 | 81.52 | 76 | 83 | 1.12 | 0.9 | 7.55 | 3.63 |
| 116 | 1508984 | good | no | 65 | no | female | left | no | no | no | no | 21.09 | 15.7 | 1.46 | 40 | 124 | 3 | 2 | 72.62 | 79.1 | 87 | 94 | 1.02 | 0.85 | 4.69 | 6.23 |
| 117 | 1510544 | good | no | 60 | no | male | left | no | no | no | yes | 23.04 | 35.8 | 1.63 | 43.3 | 140 | 4 | 1 | 84.62 | 92.48 | 86 | 92 | 1.04 | 0.93 | -5.06 | 7.85 |
| 118 | 1511551 | good | no | 61 | no | female | left | yes | yes | no | no | 22.13 | 15.8 | 1.42 | 35.5 | 119 | 2 | 3 | 77.24 | 84.76 | 72 | 84 | 1.22 | 0.86 | 7.16 | 5.23 |
| 119 | 1514963 | good | no | 75 | yes | male | left | no | no | no | no | 19.61 | 32.1 | 1.48 | 36.7 | 129 | 3 | 3 | 76.83 | 83.03 | 75 | 81 | 1.21 | 1.06 | -3.35 | 6.62 |
| 120 | 1517385 | good | no | 70 | yes | female | left | no | yes | yes | no | 28.58 | 14.5 | 1.26 | 41.2 | 130 | 1 | 1 | 75.38 | 82.41 | 73 | 82 | 0.91 | 0.71 | 7.15 | 7.44 |
| 121 | 1517828 | good | no | 70 | yes | male | left | no | yes | no | yes | 25.39 | 32.2 | 1.3 | 38.3 | 142 | 3 | 3 | 74.07 | 81.59 | 71 | 83 | 1.17 | 1.04 | 0.02 | 10.65 |
| 122 | 1518282 | bad | yes | 90 | yes | female | left | yes | yes | no | yes | 19.23 | 10.2 | 1.22 | 39.7 | 130 | 2 | 2 | 79.1 | 82.14 | 62 | 69 | 0.81 | 0.83 | 4.9 | 7.37 |
| 123 | 1522001 | good | no | 68 | no | female | right | yes | yes | no | yes | 22.48 | 20.3 | 1.17 | 40.2 | 105 | 3 | 2 | 81.45 | 90.48 | 80 | 85 | 1.34 | 0.86 | -1.85 | 9.32 |
| 124 | 1522777 | good | no | 60 | no | male | left | no | no | no | no | 20.02 | 36.5 | 1.64 | 39.7 | 133 | 2 | 3 | 82.28 | 89.66 | 85 | 93 | 0.98 | 0.83 | 5.73 | 5.58 |
| 125 | 1523409 | good | yes | 62 | no | male | right | no | no | no | no | 20.96 | 22.4 | 1.55 | 38.9 | 135 | 3 | 3 | 77.66 | 85.72 | 76 | 82 | 0.82 | 0.9 | -3.83 | 8.94 |
| 126 | 1524084 | good | no | 63 | no | female | right | no | no | yes | yes | 21.91 | 14.2 | 1.43 | 40 | 118 | 1 | 1 | 83.79 | 89.17 | 81 | 89 | 0.85 | 0.83 | 3.27 | 5.23 |
| 127 | 1524382 | good | no | 65 | no | female | left | no | no | no | yes | 22.43 | 20.1 | 1.39 | 41.9 | 122 | 1 | 3 | 77.79 | 84.69 | 76 | 82 | 0.76 | 0.72 | 9.15 | 5.12 |
| 128 | 1526241 | good | no | 65 | no | female | right | no | yes | no | no | 22.89 | 15.4 | 1.3 | 37.4 | 120 | 2 | 1 | 80.9 | 87.31 | 78 | 88 | 1.18 | 0.8 | 8.12 | 8.52 |
| 129 | 1526864 | good | yes | 66 | no | female | right | no | yes | no | no | 21.88 | 12.6 | 1.25 | 44.6 | 125 | 1 | 3 | 75.52 | 82.62 | 78 | 81 | 1.04 | 1.13 | 3.49 | 6.37 |
| 130 | 1527174 | bad | no | 61 | no | female | left | no | no | no | no | 20.82 | 18.5 | 1.34 | 33.2 | 111 | 3 | 2 | 82.48 | 88.76 | 73 | 77 | 0.85 | 1.02 | 6.48 | 7.32 |
| 131 | 1527230 | good | no | 64 | no | female | left | no | yes | yes | no | 24.03 | 21.3 | 1.34 | 33.1 | 95 | 2 | 3 | 78.69 | 86.34 | 73 | 81 | 1.18 | 1.12 | -2.82 | 8.57 |
| 132 | 1527312 | bad | yes | 74 | yes | male | left | yes | no | no | no | 25.06 | 16.4 | 1.08 | 37.3 | 139 | 2 | 3 | 68.34 | 78.28 | 66 | 72 | 0.83 | 1.05 | 6.44 | 16.79 |
| 133 | 1530289 | good | no | 63 | no | female | right | no | no | no | no | 19.78 | 18.2 | 1.2 | 34.6 | 116 | 2 | 3 | 85.17 | 90.62 | 88 | 95 | 1.04 | 0.75 | 7.4 | 10.51 |
| 134 | 1531547 | good | no | 62 | no | male | right | no | no | no | no | 22.6 | 36.5 | 1.66 | 39.8 | 128 | 2 | 3 | 81.59 | 86.28 | 86 | 91 | 1.02 | 0.83 | 7.99 | 9.38 |

| Number | Patient ID | Functional outcome | Sarcopenia | Age | ⩾ 70y | Sex | Affect side | Cardiac disease | Hypertensive disease | Diabetes | Hyperli-pidemia | Bmi | Grip strength | tmCSA/BW | Albumin | Hemoglobin | Wait time for surger | AO | R3 | R6 | HSS 3 | HSS 6 | IS | BP | CA | LFA |
|---|---|---|---|---|---|---|---|---|---|---|---|---|---|---|---|---|---|---|---|---|---|---|---|---|---|---|
| 135 | 1531080 | good | no | 65 | no | female | right | no | yes | no | yes | 19.53 | 14.8 | 1.66 | 37.2 | 125 | 1 | 3 | 83.59 | 88.9 | 91 | 98 | 1.16 | 1.05 | -2.46 | 4.18 |
| 136 | 1530910 | good | yes | 60 | no | male | right | no | no | no | yes | 23.24 | 27.3 | 1.14 | 36.4 | 165 | 2 | 3 | 79.17 | 83.03 | 80 | 84 | 0.92 | 0.69 | 5.07 | 11.17 |
| 137 | 1534317 | good | no | 70 | yes | female | left | yes | no | no | no | 25.39 | 17.5 | 1.42 | 37 | 127 | 1 | 1 | 72.9 | 78.83 | 75 | 85 | 0.91 | 0.83 | -3.58 | 5.41 |
| 138 | 1535519 | good | yes | 61 | no | male | left | no | no | yes | yes | 19.72 | 21.1 | 1.54 | 32.2 | 126 | 1 | 2 | 82.9 | 90.83 | 81 | 82 | 0.92 | 0.96 | 3.15 | 4.27 |
| 139 | 1536787 | good | no | 74 | yes | male | left | no | no | no | no | 24.82 | 28.5 | 1.35 | 33.3 | 167 | 2 | 3 | 75.59 | 79.86 | 76 | 84 | 1 | 0.88 | 5.31 | 9.04 |
| 140 | 1537753 | good | no | 63 | no | female | left | yes | yes | no | no | 22.23 | 16.4 | 1.54 | 47.9 | 142 | 1 | 3 | 79.17 | 86 | 86 | 92 | 0.89 | 1.12 | 4.58 | 7.13 |
| 141 | 1540462 | good | no | 64 | no | male | left | no | yes | no | yes | 24.22 | 32.7 | 1.62 | 44.1 | 174 | 1 | 3 | 80.83 | 84.48 | 89 | 94 | 0.92 | 0.93 | 3.51 | 13.25 |
| 142 | 1540848 | good | no | 64 | no | male | left | no | no | no | no | 17.76 | 34.5 | 1.88 | 33.9 | 147 | 2 | 3 | 87.1 | 92.97 | 91 | 96 | 1.17 | 0.8 | -2.62 | 6.52 |
| 143 | 1542701 | good | no | 60 | no | male | left | no | yes | no | yes | 29.4 | 30.8 | 1.25 | 40 | 143 | 4 | 2 | 77.17 | 83.72 | 94 | 98 | 0.9 | 0.77 | 10.06 | 4.59 |
| 144 | 1543711 | good | no | 60 | no | male | left | yes | yes | no | no | 22.86 | 23.2 | 1.59 | 41.2 | 151 | 4 | 3 | 80.97 | 87.38 | 93 | 96 | 1.04 | 0.96 | 4.88 | 4.69 |
| 145 | 417095 | good | no | 65 | no | female | left | no | no | no | no | 22.58 | 21.7 | 1.35 | 41.6 | 127 | 1 | 1 | 72.07 | 78.07 | 87 | 91 | 0.76 | 1.14 | 4.71 | 8.52 |
| 146 | 417532 | good | no | 61 | no | female | left | no | no | no | no | 20.55 | 20.9 | 1.23 | 42.6 | 120 | 2 | 2 | 84.07 | 89.31 | 85 | 93 | 0.94 | 0.82 | -3.97 | 8.87 |
| 147 | 418395 | bad | yes | 67 | no | female | left | no | no | no | no | 20.96 | 11.4 | 1.17 | 41.8 | 119 | 3 | 3 | 77.17 | 84.55 | 69 | 75 | 0.89 | 1.11 | -3.02 | 7.3 |
| 148 | 425560 | good | no | 61 | no | male | left | yes | no | no | no | 22.49 | 26.3 | 1.66 | 40.3 | 126 | 5 | 3 | 81.1 | 87.1 | 75 | 84 | 1.19 | 0.84 | 6.58 | 5.75 |
| 149 | 427804 | good | no | 67 | no | male | left | no | no | no | yes | 27.28 | 28.3 | 1.63 | 48 | 155 | 3 | 2 | 78.48 | 86.14 | 78 | 86 | 1.04 | 0.84 | -0.89 | 8.27 |
| 150 | 430304 | bad | yes | 67 | no | male | right | no | yes | no | no | 23.03 | 21.1 | 1.36 | 39.5 | 147 | 2 | 3 | 71.24 | 77.86 | 69 | 71 | 1.1 | 0.95 | 4.9 | 6.5 |
| 151 | 430879 | good | no | 60 | no | male | right | no | no | yes | no | 22.23 | 35.2 | 1.64 | 43.6 | 142 | 4 | 3 | 77.72 | 87.45 | 95 | 98 | 0.97 | 0.79 | -3.83 | 8.87 |
| 152 | 437320 | good | yes | 69 | no | female | left | no | no | no | no | 23.44 | 13.5 | 1.24 | 42.6 | 122 | 2 | 1 | 72.69 | 77.52 | 70 | 84 | 0.9 | 1.14 | -2.94 | 7.2 |
| 153 | 437321 | bad | no | 84 | yes | female | left | yes | yes | no | no | 29.14 | 15.8 | 1.27 | 43.3 | 124 | 2 | 3 | 68.07 | 78.07 | 68 | 72 | 1.03 | 0.76 | 4.58 | 7.74 |
| 154 | 439368 | good | no | 62 | no | female | left | no | no | yes | yes | 25.16 | 23.4 | 1.21 | 42.3 | 121 | 4 | 3 | 80.62 | 87.31 | 93 | 94 | 1.09 | 1.12 | 5.81 | 12.78 |
| 155 | 441876 | good | no | 62 | no | female | left | no | no | no | no | 21.48 | 18.3 | 1.31 | 44.5 | 126 | 1 | 3 | 83.59 | 87.66 | 85 | 89 | 0.89 | 1.02 | -1.8 | 10.49 |
| 156 | 443354 | good | no | 60 | no | male | right | no | no | no | no | 26.99 | 36.4 | 1.6 | 41.4 | 136 | 1 | 3 | 79.86 | 82.28 | 87 | 93 | 0.95 | 0.77 | -3.39 | 8.38 |
| 157 | 452980 | bad | no | 67 | no | female | right | no | no | yes | no | 27.06 | 19 | 1.24 | 39.2 | 111 | 1 | 2 | 83.66 | 92.07 | 73 | 80 | 0.81 | 0.83 | 5.55 | 8.14 |
| 158 | 454450 | good | no | 60 | no | female | left | no | no | no | no | 26.67 | 25.3 | 1.31 | 43.2 | 125 | 1 | 1 | 70.69 | 79.72 | 86 | 90 | 0.91 | 0.99 | 7.68 | 16.83 |
| 159 | 456742 | bad | no | 70 | yes | male | right | no | yes | no | yes | 20.9 | 31.2 | 1.62 | 40.3 | 148 | 4 | 3 | 80.41 | 84.76 | 80 | 80 | 0.93 | 0.7 | -4.87 | 11.51 |
| 160 | 469428 | good | no | 81 | yes | male | right | no | yes | no | no | 29.37 | 34.2 | 1.62 | 37.4 | 123 | 3 | 3 | 74.62 | 80.97 | 74 | 82 | 1.06 | 0.94 | -5.39 | 9.27 |
| 161 | 469431 | good | yes | 61 | no | male | right | no | yes | no | no | 19.14 | 22.7 | 1.54 | 38.9 | 124 | 3 | 3 | 85.17 | 89.31 | 79 | 82 | 1.16 | 1.08 | 5.51 | 4.96 |
| 162 | 472720 | good | no | 64 | no | male | left | no | no | no | no | 23.64 | 31.1 | 1.69 | 44.4 | 127 | 1 | 3 | 83.03 | 90.14 | 91 | 94 | 0.92 | 0.72 | -3.84 | 11.71 |
| 163 | 478338 | good | no | 67 | no | female | right | no | yes | no | no | 19.53 | 20.4 | 1.23 | 43.7 | 110 | 3 | 3 | 88.34 | 92.97 | 88 | 91 | 0.94 | 0.94 | 5.02 | 5.93 |
| 164 | 480338 | bad | yes | 64 | no | female | right | no | yes | no | no | 19.53 | 14.5 | 1.21 | 44.4 | 115 | 3 | 2 | 88.55 | 92.83 | 71 | 78 | 0.91 | 0.71 | 5.27 | 7.32 |
| 165 | 483590 | bad | no | 77 | yes | male | right | yes | no | no | no | 24.21 | 24.2 | 1.65 | 38.2 | 132 | 1 | 2 | 74.14 | 81.59 | 72 | 79 | 0.93 | 0.84 | 3.47 | 6.18 |
| 166 | 485568 | good | no | 70 | yes | male | right | no | yes | no | yes | 27.68 | 23.9 | 1.59 | 42.6 | 134 | 5 | 3 | 71.59 | 76.21 | 76 | 84 | 1.15 | 0.96 | 0.27 | 5.83 |
| 167 | 486498 | bad | yes | 76 | yes | male | left | yes | yes | no | no | 22.59 | 15.3 | 1.47 | 40.1 | 134 | 1 | 3 | 68.48 | 73.17 | 63 | 65 | 1.14 | 1.21 | -5.69 | 10.12 |
| 168 | 485605 | good | no | 60 | no | female | left | no | no | yes | yes | 19.11 | 21.6 | 1.78 | 40.9 | 118 | 6 | 3 | 85.31 | 91.86 | 87 | 91 | 1.25 | 0.96 | -1.53 | 7.49 |

IS(Insall-Salvati index)
BP(Blackburne-Peel index)
CA(Congruence angle)
LFA(Lateral patellofemoral angle)
